# Plant science in the age of simulation intelligence

**DOI:** 10.3389/fpls.2023.1299208

**Published:** 2024-01-16

**Authors:** Michiel Stock, Olivier Pieters, Tom De Swaef, Francis wyffels

**Affiliations:** ^1^ KERMIT and Biobix, Department of Data Analysis and Mathematical Modelling, Ghent University, Ghent, Belgium; ^2^ IDLAB-AIRO, Ghent University, imec, Ghent, Belgium; ^3^ Plant Sciences Unit, Flanders Research Institute for Agriculture, Fisheries and Food, Melle, Belgium

**Keywords:** simulation intelligence, digital agriculture, phenotyping, quantified plant, digital twin, modeling, scientific computing, artificial intelligence

## Abstract

Historically, plant and crop sciences have been quantitative fields that intensively use measurements and modeling. Traditionally, researchers choose between two dominant modeling approaches: mechanistic plant growth models or data-driven, statistical methodologies. At the intersection of both paradigms, a novel approach referred to as “*simulation intelligence*”, has emerged as a powerful tool for comprehending and controlling complex systems, including plants and crops. This work explores the transformative potential for the plant science community of the nine simulation intelligence motifs, from understanding molecular plant processes to optimizing greenhouse control. Many of these concepts, such as surrogate models and agent-based modeling, have gained prominence in plant and crop sciences. In contrast, some motifs, such as open-ended optimization or program synthesis, still need to be explored further. The motifs of simulation intelligence can potentially revolutionize breeding and precision farming towards more sustainable food production.

## 1 Introduction

As computational capabilities have grown, modeling has become a specialized area of agricultural sciences. These models can accurately simulate the performance of plants, crops, and greenhouses under various conditions and have therefore been used extensively for generating scientific hypotheses, informing and accelerating breeding programs, optimizing crop management and providing policy recommendations (Silva and Giller, 2020). However, models remain highly species-specific, complex, and difficult to calibrate due to many interlinked parameters (Wallach et al., 2021). Current developments and increased availability of phenotyping data provide an extensive source of data for model development and calibration, required for the extension of model applicability to novel or ‘forgotten’ crops and to studying impacts of pests and micronutrients. (Silva and Giller, 2020). However, more modern and powerful modeling paradigms are needed to address these issues and to infer large sets of parameters from phenotyping data. This paper introduces some fundamental concepts of the emerging field of *simulation intelligence* (SI) to plant science. SI is the merger of scientific computing and artificial intelligence (Lavin et al., 2021). Specifically for plant science, this will result in a new field that combines novel phenotyping approaches with modeling.

Phenotyping is quantifying (a subset of) plant traits that result from the interaction between plant genetics and the environmental conditions to which plants are exposed (Walter et al., 2015). Due to internal regulatory mechanisms, these phenotypic responses are situated at multiple organizational scales (cell, tissue, organ, plant, field) and across timescales. Biotic and abiotic external drivers also influence these mechanisms. While phenotyping ideally involves capturing the entire state of a plant in space and time, only partial observations are practically feasible, leading to the need for a wide range of sensory devices that capture part of the phenotype. A more holistic and dynamic view of phenotyping is necessary to overcome challenges involved in current approaches, including improving the temporal resolution and broadening results from specific studies to more diverse conditions (Das Choudhury et al., 2018).

Most models consist of mathematical equations for predicting plant behavior, morphology and growth as a function of environment, genetics, and management. Plant models describe and connect plant processes typically studied in isolation and consequently predict integrated responses. As such, models are often used for hypothesis development and improved understanding of plant processes, but also as decision support tools for breeding (e.g., genomic prediction), crop management (e.g., irrigation scheduling) and policy-making (e.g., climate change scenarios) (Peng et al., 2020).

Depending on their objective, models vary at the level with which processes are included (black-box ↔ mechanistic axis) and at the scale they operate in terms of space (molecule ↔ ecosystem axis) and time (second ↔ century axis). Models are considered ‘process-based’ or ‘mechanistic’ when parameters have a biophysical interpretation and their equations explicitly describe processes (e.g., photosynthesis, water transport). They operate at a different spatial and (often also) temporal scale. At each spatial level, there is an extra level of abstraction, but, interestingly, the scale does not necessarily determine whether a model is more or less mechanistic, as plants tend to adapt to their environment in an integrated way (Tardieu et al., 2020). On the other end of the spectrum, entirely data-driven models based on machine learning algorithms often lack interpretable parameters. The latter group of models is vital in breeding [e.g., genomic prediction (Korte and Farlow, 2013; Hickey et al., 2017; De Meyer et al., 2023)], but also in greenhouse climate control (Hemming et al., 2020). Consequently, models often only operate on a single point in the tempo-spatial domain, limiting their use beyond their initial conceptualization. Nevertheless, there are efforts to connect modeling scales from the molecular level up to the crop system (Peng et al., 2020).

Recently, surrogate plant models have become popular (Corrales et al., 2022; Cheng et al., 2023; Zhang et al., 2023), because these allow for creating a “digital twin” of the plant systems for decision support and control. A surrogate data-driven model is trained to mimic the mechanistic model’s output accurately. When properly trained, such a surrogate can be several magnitudes faster than the original model while behaving nearly identically (Gherman et al., 2023). Apart from the complete replacement of mechanistic models by data-driven models, these can also be combined. For example, Zhang et al. (2023) demonstrate how these can be coupled in series (e.g., crop model simulation outputs as input to a machine learning model), in parallel (e.g., data assimilation in crop models) or via modules (e.g., part of a crop model is replaced by a machine learning module). This is a stepping stone towards SI, leading to cross-pollination between scientific computing, artificial intelligence, plant modeling and phenotyping.

## 2 Combining scientific computing with artificial intelligence

Complex biological systems require powerful tools to study them. On the one hand, many of these systems require substantial domain knowledge, often as conservation laws and reaction mechanisms, for which traditional mechanistic modeling and simulation paradigms are well suited. This is known as “scientific computing” and relies mainly on ordinary differential equations, partial differential equations, agent-based models and their ilk. On the other hand, many mechanisms are yet to be elucidated while, at the same time, a plethora of multimodal data is available. This motivates using a data-driven approach (referred to as “artificial intelligence” or “machine learning”). Recent advances blur the lines between traditional methodologies, and so-called *scientific machine learning* combines both, for example, in neural ordinary differential equations (Chen et al., 2018; Innes et al., 2019; Rackauckas et al., 2021), where the solvers are treated as differentiable programs that can fit data to learn unknown dynamics of the problems.

**Figure 1:**
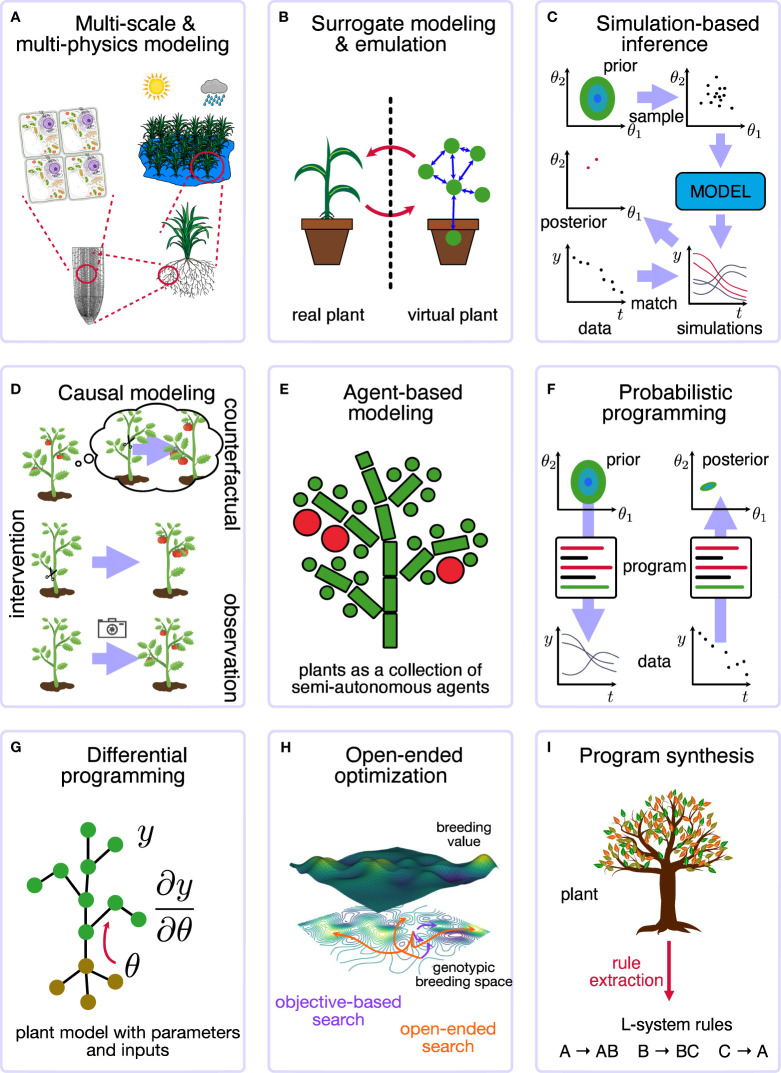
Overview of the different SI motifs for plants. **(A) Multi-scale and multi-physics modeling** considers the different scales (from cell to ecosystem or field) and physical processes (radiation, hydraulics, fluidics). **(B) Surrorgate modeling and emulation** considers a virtual digital twin of the plant system. **(C) Simulation-based inference**, such as approximate Bayesian computing, allows a simulator to infer parameters or states from data. **(D) Causal modeling and inference** takes into account the different levels of causal reasoning that are possible. **(E) Agent-based modeling** simulates a system as a collection of semiautonomous agents. **(F) Probabilistic programming** allows for general computing with stochastic components and performs general inferences about parameters and states. **(G) Differential programming** computing and simulation with gradients and derivatives. **(H) Open-ended optimization** aims at finding continuous improvements and adaptations, for example, in plant breeding. **(I) Program synthesis** automatically generates the code to solve a problem, e.g., extract the L-system to describe a plant.

The advances in scientific computing and machine learning and their use in studying complex, dynamical multi-scale systems gave rise to a more generalized view: the new field of *simulation intelligence* (SI). Lavin et al. (2021) outlined nine vital, interconnected computing technology motifs, visually represented in **Figure 1**:

1. **Multi-scale and multi-physics modelling** (Karniadakis et al., 2021): integrating different types of simulators;
2. **Surrogate modelling and emulation** (Purcell and Neubauer, 2023): replacing a complex model or system with a different one;
3. **Simulation-based inference** (Cranmer et al., 2020): using the simulator to infer parameters or states;
4. **Causal modelling and inference** (Schölkopf et al., 2021): including or identifying causal concepts within the model;
5. **Agent-based modelling** (Zhang and DeAngelis, 2020): simulating a system as a collection of semiautonomous agents;
6. **Probabilistic programming** (Schoot et al., 2021): interpreting code as a stochastic program;
7. **Differentiable programming** (Baydin et al., 2018): computing and using derivatives and gradients of computer code and simulators;
8. **Open-ended optimization** (Stock and Gorochowski, 2023): trying to find continuous improvements;
9. **Program synthesis** (David and Kroening, 2017): automatically discovering the code to solve a problem.

We included a reference for each motif that covers this specific topic more in-depth.

This technology stack has enormous potential to advance fields such as material science, agriculture, chemistry, medicine, climate, and synthetic biology. Simulation intelligence can also significantly impact plant sciences. By combining modeling and phenotyping, one can uncover mechanisms underlying plant dynamics. For example, functional-structural plant modeling aims to develop holistic plant growth and function models, harmonizing architecture with (eco)physiology. The Quantitative Plant^1^ initiative collects plant phenotyping data sets, plant analysis tools and models. The availability of plant phenotypic data repositories and plant biophysical models are prerequisites for SI.^1^

Concretely, SI aims to handle the following challenges in using computational modeling for real-world problems:

- **Inverse problem solving**, where one wants to use a model to infer hidden states or parameters from observations or measurements. For example, in root phenotyping, researchers use electrical resistance tomography measurements to infer root properties non-invasively (Whalley et al., 2017).
- **Uncertainty reasoning**, which relates to the inherent uncertainty of dealing with biological systems, both epistemic (i.e., incomplete knowledge of the processes) and aleatoric (i.e., the irreducible noise, for example, due to biological stochasticity) (Hüllermeier and Waegeman, 2021). Quantifying uncertainty is of great importance for plant breeding and precision crop management (Asseng et al., 2013; Tao et al., 2018; Folberth et al., 2019; Nelson et al., 2019; Hernández and López, 2020; Dokoohaki et al., 2021), especially when dealing with a changing climate.
- **Human-machine teaming** relates to the interaction between the model or machine intelligence and the breeder, farmer or other users. This includes intelligent dashboards and ways for users to query the simulator for decision-making and inject data, observations and results into the model. Bridging the gap between models and users is a significant challenge in digital agriculture (Antle et al., 2017; Slob et al., 2023; Zhang et al., 2023).

## 3 Nine simulation intelligence motifs for plant science

This section discusses the nine SI motifs outlined in Lavin et al. (2021) and discusses how they can relate to plant science. We speculate about the opportunities they could present in digital agriculture when we find only a few plant-related examples. Due to the broad scope of these topics, we have to be concise. Our primary aim is to inform the quantitative plant scientist of available SI tools. We refer to the work of Lavin et al. (2021) or specific overview papers for an extensive introduction.

### 3.1 Multi-scale and multi-physics modeling

Plant processes occur on different scales (**Figure 1A**). These can be spatial, from the molecular processes in the cell (micrometers) to the ecosystem (up to kilometers), or temporal, the processes of interest range from nanoseconds (e.g., photosynthesis) to months or years (e.g., growth). For example, plants generate and use various rhythms and oscillations at all scales and organization levels (Damineli et al., 2022). Plant modelers are aware of the fractal complexity of plant modeling, where lower-level processes can be abstracted away in so-called *meta-mechanisms* (Tardieu et al., 2020). Meta-mechanisms are, e.g., response curves of plant traits to environmental conditions, which can be characterized in a high-throughput fashion. Such meta-mechanisms are largely determined by physical trade-offs that limit evolution (Kempes et al., 2019). Meta-mechanisms can be tailored to specific plant species or cultivars, an open challenge in plant modeling (Silva and Giller, 2020).

Plant models involve various kinds of physical models, going from molecular and metabolic processes (Farquhar et al., 1980), hydraulic functioning (De Swaef et al., 2022), to soil and atmospheric physics (Liu et al., 2020b). Modern (functional-structural) plant modeling involves advanced physics simulation such as ray tracing to assess radiation (De Visser et al., 2014; Bailey, 2018; Retkute et al., 2018) and computational fluid dynamics (Bartzanas et al., 2013; Jiao et al., 2020). The latter are often computationally demanding and might require appropriate tools, such as surrogate modeling (discussed later), to make them feasible for, e.g., greenhouse control.

Physics-informed machine learning can be a powerful aid in incorporating the different scales and physics (Karniadakis et al., 2021). Here, data-driven models are fitted not only to match their training data but also to adhere to known physical laws and are ideally suited to integrate data into different physical processes. This has shown great success in biomedical modeling, for example, in modeling blood flow in an intracranial aneurysm (Raissi et al., 2020). In crop science, Cavanagh et al. (2021) used physics-informed deep learning to study the morphological changes induced by Asian soybean rust. Similar directions for holistic plant modeling will undoubtedly be fruitful.

### 3.2 Surrogate modeling and emulation

A *surrogate model* is a model that replaces an often expensive computation or process (**Figure 1B**). In scientific computing, expensive simulations, such as computational fluid dynamics, are often replaced by relatively inexpensive methods of training and deploying machine learning surrogates, such as Gaussian processes or artificial neural networks. For example, Cheng et al. (2023) used a data-driven surrogate model in combination with a multi-objective genetic algorithm to reduce irrigation and nitrogen fertilization by 44% and 37%, respectively.

Surrogate models play a pivotal role in developing of *digital twins* – real-time synchronized virtual representations of products, processes, or environments. These dynamic digital counterparts facilitate a bidirectional flow of information, leveraging real-world data while influencing management and decision-making processes. Positioned at the forefront of digital agriculture and smart farming (Verdouw et al., 2021; Slob et al., 2022; Purcell and Neubauer, 2023), digital twins seamlessly integrate with the principles of Industry 4.0 tailored for agricultural contexts.

Digital twins exhibit versatility, being applied to emulate plants, greenhouses, or entire supply chains. Their primary utility is improving cost-efficiency, such as reducing water and fertilizer consumption and elevating prediction accuracy (Ariesen-Verschuur et al., 2022). Verdouw et al. (2021) categorizes digital twins based on their relationship to virtual objects—whether it pertains to an imaginary entity (e.g., a yet-to-grow cultivar), an existing object, future states for predictive analysis, or a historical object. Additionally, digital twins serve distinct purposes, being employed for both monitoring and prescription.

In recent years, digital twins have demonstrated significant successes in agriculture. Examples include emulating various wheat development stages to predict yield (Skobelev et al., 2020), optimizing yield and minimizing energy requirements in underground hydroponic farms (Jans-Singh et al., 2020), exploring virtual replicas of greenhouses through immersive VR experiences (Slob et al., 2023), monitoring the health and quality of individual plants in orchards (Moghadam et al., 2020), and fine-tuning greenhouse control systems (Chaux et al., 2021). Using simulations to govern systems and explore hypothetical interventions aligns closely with causal reasoning, as discussed in Section 3.4.

### 3.3 Simulation-based inference

In plant modeling, knowledge of the processes of interest is often encoded in process-based models. Typically, given the initial conditions and the parameter values, these models can simulate data that can be compared with quantitative measurements such as biomass growth, development stage, or transpiration. Generating this data is called forward modeling. However, when one observes the data, one would often infer the likely hidden states or parameter values, a process that is a much more challenging inverse problem (**Figure 1C**). The field of simulation-based inference deals with developing inference methods for highly intricate simulators, i.e., to extract the parameters of a mechanistic model algorithmically (Cranmer et al., 2020). Simulation-based inference is often called *likelihood-free inference* – as contrasted with classical statistical estimation problems. The likelihood function implicitly defined by the simulator is often not tractable, making this a challenging endeavor. Inverse problems are usually solved using a Bayesian perspective, where the parameters or states have associated prior distributions. The simulator acts as an implicit likelihood function, linking the model with the data and parameters.

Approximate Bayesian Computation (ABC) is a widely utilized approach for simulation-based inference (Marin et al., 2012; Romero-Cuellar and Francés, 2023). In ABC, the simulator generates synthetic data by sampling parameters from a prior distribution or proposal distribution and using these parameters to perform a simulation. These synthetic datasets are characterized by summary statistics, such as the total biomass, used to compare the simulated data with collected observations. Parameter values producing synthetic data with summary statistics closely aligned with those of the actual data, often measured using Euclidean distance, are retained. These selected values provide approximate samples of the posterior distribution. ABC’s most commonly used variant operates similarly to rejection sampling, and its sampling properties are well understood. However, the conventional ABC method becomes inefficient, especially when dealing with large parameter spaces. Notably, ABC has been successfully applied in plant science to merge crop growth models with whole genome data (Technow et al., 2015), to infer root architecture (Ziegler et al., 2019) and to characterize the morphodynamic progression of Asian soybean rust (Cavanagh et al., 2021). The progression of machine learning and SI techniques, including probabilistic and differentiable programming (see Section 3.5), has significantly influenced simulation-based inference. For instance, in a study by Monti et al. (2023), the parameters of an agent-based model were learned directly from data by redefining it as a probabilistic program.

### 3.4 Causal modeling and inference

Data-driven modeling has achieved remarkable success across various scientific and technological domains. However, purely statistical models often fail to uncover the underlying causal mechanisms behind the observed data. As an illustration, consider a simple linear regression model predicting yield based on nutrient inputs. This model might erroneously suggest that fertilization decreases yield, neglecting the confounding effect of poorer soils where fertilizers are commonly applied.

The significance of understanding causality has been underscored by Judea Pearl in his work, including “The Book of Why” (Pearl and Mackenzie, 2018). Pearl introduced a hierarchy of causal reasoning that data-driven models can accomplish, comprising:

1. **Observations:** Detecting associations in data, such as estimating tree biomass from their diameter at breast height.
2. **Interventions:** Predicting outcomes resulting from active manipulations of the system, like projecting the effects of flower pruning on fruit production.
3. **Counterfactuals:** Imagining potential scenarios where conditions or interventions differed, as in assessing whether larger fruits would have resulted from more extensive flower removal.

Pearl’s mathematical insights reveal that some models are inherently limited in performing higher-level causal reasoning. Thus, plant scientists who aim to predict and manage must exercise caution when employing data-driven models from observational data because the causal link between the predictors is not exploited by default. For example, a data-driven model might conclude that watering harms a plant’s water status, as irrigation and water stress are correlated. This scenario highlights the limitations of relying solely on observational data, representing the first level of causal reasoning in Judea Pearl’s hierarchy, where associations in data are detected without considering active manipulations or counterfactual scenarios. The limitations contrast with many mechanistic models, which can often be used directly for interventions and counterfactuals. The crux lies in developing models incorporating the structural relationships between variables of interest, advocating for mechanistic and hybrid models. The evolving field of causal machine learning continues to gain prominence (Schölkopf et al., 2021) (**Figure 1D**).

### 3.5 Agent-based modelling

*Agent-based models* (ABMs) depict complex systems as interconnected, autonomous agents (e.g., organs or whole plants) interacting from the bottom up (**Figure 1E**). These models frequently encompass stochastic elements and can replicate macro-level processes stemming from micro-level interactions. Consequently, ABMs are a natural fit for elucidating multi-scale phenomena. In ecology and plant science, ABMs are widely employed (DeAngelis and Mooij, 2005; McLane et al., 2011; Zhang and DeAngelis, 2020), offering insights into growth, carbon allocation, reproduction, and more. These models can portray individual plants within functional-structural plant models (FSPMs) or capture entire plant communities, such as field ecosystems.

Interestingly, ABMs precisely capture individual plant behaviors due to plants’ modular structure, comprising elements like roots, leaves, stems, and branches. Each module functions autonomously, gathering, producing, or distributing resources for the overall plant’s advantage. Remarkably, plants lack a central controlling entity, resembling a decentralized “swarm intelligence” (Baluška et al., 2010a; Oborny, 2019; van Schijndel et al., 2022). For instance, a plant’s root tips exhibit both sensory and command center roles, independently deciding growth directions and even forming symbiotic relationships with mycorrhizal fungi (Baluška et al., 2010b). Some liken this to a “solid” brain, where individual units are fixed. In contrast, others argue for “liquid” brain aspects (van Schijndel et al., 2022), like in plants with vegetative propagation, like strawberries, exploring diverse niches to optimize their niche.

### 3.6 Probabilistic programming

The language of probability theory is an effective way to describe biological systems, given their inherent stochastic nature (**Figure 1F**). Specifically, Bayesian statistics is a consistent framework to update the scientist’s prior beliefs (encoded in prior distributions) with measurements and observations (encoded in the likelihood) into the so-called posterior distribution (Schoot et al., 2021). In plant science, Bayesian reasoning is applied in, for instance, plant pathology and epidemics (Mila and Carriquiry, 2004), modeling life stage events (Humplík et al., 2020), and predicting maize yield (Lacasa et al., 2020). Though powerful, Bayesian and probabilistic methods can be complex in practice because conditioning a distribution (e.g., computing the posterior) requires normalization, often involving computing intractable integrals or sums. Probabilistic programming is a relatively new, general approach to making probabilistic methods more accessible in the scientific community.

A *probabilistic programming language* (PPL) allows one to write, in principle, arbitrary complex stochastic programs from which the scientist can make inferences by sampling. Hence, a universal PPL provides two constructs: i) a way to sample from the stochastic program and ii) a way to condition during inference. For example, one can write a program to simulate flowering vines and then constrain regions where they are present. This allows one to sample vines that grow in a specific shape, such as a letter (Ritchie et al., 2016). PPLs have shown success throughout the biological sciences, for example, in inferring phylogeny (Ronquist et al., 2020), protein structure alignment (Moreta et al., 2019) and inferring signaling pathways (Merrell and Gitter, 2020). There are a plethora of PPLs available, many interfacing with scientifically popular programming languages for sciences, for example, Stan (Stan Development Team, 2023), Pyro (Bingham et al., 2019), or Turing (Holt and Cordy, 1988).

### 3.7 Differentiable programming

While probabilistic programming facilitates generic computations involving probability distributions, *differentiable programming* (Izzo et al., 2016; Innes et al., 2019) extends computation by enabling differentiation of arbitrary computer programs (**Figure 1G**). This empowers the fine-tuning of program behavior using gradient-based optimization techniques. This achievement relies on *automatic differentiation* (Baydin et al., 2018) – numerically computing (exact) derivatives by directly manipulating the computational graph – a foundational concept in deep learning. Differentiable programming has exerted a profound scientific influence, acting as a cornerstone for nearly all deep learning research over the past decade and diverse domains beyond deep learning. These domains encompass ordinary differential equations (Chen et al., 2018; Rackauckas et al., 2021; Núñez et al., 2023), scientific machine learning, robotics Degrave et al. (2019), physics, protein science (Ingraham et al., 2019; AlQuraishi and Sorger, 2021), combinatorial optimization (Liu et al., 2020a), and geosciences (Shen et al., 2023). The utility of differentiable programming extends to harmonizing process-based and data-driven models. Within plant sciences, differentiable plant models offer an avenue to assess sensitivity directly, calibrate parameters using gradients, apply probabilistic programming techniques for uncertainty quantification, and gain control over conditions for optimizing growth. Concrete achievements in plant sciences include the creation of 3D digital twin leaf models from image data (Li et al., 2022) and solving inverse problems related to photosynthesis (Aboelyazeed et al., 2023). We also propose that advancements in differentiable ray tracing (Li et al., 2018), computational fluid dynamics (Bezgin et al., 2023), and physics engines (de Avila Belbute-Peres et al., 2018; Degrave et al., 2019) hold substantial promise for enhancing plant simulations.

### 3.8 Open-ended optimization

Open-ended systems possess the ability to achieve limitless improvement and continuously generate novelty (Stanley and Lehman, 2015; Banzhaf et al., 2016; Stanley et al., 2017) (**Figure 1H**). In such systems, the focus primarily lies on creating novelty rather than being driven by a specific objective function (Stanley and Lehman, 2015). Open-endedness is a characteristic observed in various complex systems, including natural evolution and technological innovation. Its principles have been explored for diverse applications such as designing new computer architectures (Ackley and Small, 2014), software development (Fix et al., 2021), artificial neural networks (Guttenberg et al., 2018), and novel cancer treatment strategies (Balaz et al., 2021).

Our other work delves into how open-endedness and quality-diversity algorithms can contribute to biotechnology and synthetic biology (Stock and Gorochowski, 2023). Expanding this perspective, we propose that open-endedness can significantly impact plant breeding, a critical aspect in ensuring global food security (Lenaerts et al., 2019). Conventional breeding approaches often prioritize incorporating positive traits into populations, potentially at the expense of diversity (Louwaars, 2018). A noteworthy exception occurred in the 1970s when Zelder, a breeding company, intentionally bred wheat varieties to enhance diversity as a defense against yellow rust (Groenewegen, 1977). Embracing the open-ended optimization viewpoint, one could design breeding schemes capable of continually generating new cultivars with novel and desirable traits. Insights from the field of quality-diversity optimization (Pugh et al., 2016), which focuses on generating new variants that combine functionality and diversity, have the potential to revolutionize breeding strategies for developing crops and cultivars suited to a dynamically changing world. In silico evolution experiments can help to understand the allometric relations observed in plants due to environmental conditions (Eloy et al., 2017). As such, they might help to design new cultivars.

### 3.9 Program synthesis

*Program synthesis* automates software creation to tackle specific problems (David and Kroening, 2017) (**Figure 1I**). Here, the focus shifts towards generating optimized code by capturing users’ intentions. A notable application of this concept is evident in the recently released ChatGPT, where users use natural language queries to program or create computer code. This synthesis technique plays a pivotal role in simplifying intricate mathematical system descriptions.

Program synthesis offers avenues for extracting insights from biological experiments in mathematical modeling. For instance, Koksal et al. (2013) automatically generated biological models from mutation experiments and recommended new experiments to differentiate between potential models. This approach holds promise for analyzing high-throughput CRISPR-Cas-based knockout experiments, offering valuable insights for plant breeding (Van Huffel et al., 2022). Symbolic regression uses genetic programming to automatically discover a white-box model of one system (Angelis et al., 2023; Cranmer, 2023) – ideal to find the earlier-discussed meta-mechanisms. The DreamCoder system can uncover simple programs that generate example datasets (Ellis et al., 2023). These programs encompass diverse forms, such as regular expressions, graphics, symbolic equations, and physical laws. These techniques aid in discovering allometric laws and meta-mechanisms to support model building. They also facilitate the automatic extraction of rules for L-systems, enabling the creation of virtual plants based on a limited set of examples. In summary, custom computer algebra systems (Ma et al., 2022) and language compilers can streamline equations and code, resulting in concise and numerically stable plant models.

## 4 Discussion and outlook

The sections above discussed SI and its (potential) impact on the plant sciences. Here, we will give a more holistic point of view of why SI can be instrumental in discovering new and improving current practices in plant sciences. SI provides a holistic, top-down look at plant science and a systemic approach for leveraging fragmented phenotypic data and ecophysiological knowledge contained in process-based models.

Process-based plant models (including FSPMs) are continuously under development and updated with relevant knowledge. These are applied for decision support and (climate) scenario analysis, but also for answering scientific, plant-physiological questions, often in combination with plant phenotypic data. High-throughput plant phenotyping data of crop performance and development is now also being applied in crop breeding (Araus and Cairns, 2014; Gill et al., 2022). Still, the impact of these phenotypic data often needs to be more specific to the objectives of the experiments wherein these were collected. Therefore, SI concepts can facilitate the connection between phenotypic data and ecophysiological plant models and, as such, broaden the use of these phenotypic data and expand knowledge of the processes they rely on.

We identify three key prerequisites to embrace SI’s philosophy in plant sciences fully. (1) The development of cheap sensor technology for environmental monitoring and plant phenotyping enables continuous monitoring of larger populations in real-time. (2) Open datasets and code, following the FAIR principles (Wilkinson et al., 2016), which is finding its way into plant sciences (Saint Cast et al., 2022) but is already much more prevalent in other scientific domains (Scheffler et al., 2022). (3) Interdisciplinary collaborations bridge potential knowledge gaps and enable cross-disciplinary approaches to succeed faster.

The increased amount of open data, along with AI, allows the processing of larger amounts of combined data and models and opens up new or improved applications in plant breeding or greenhouse control, as seen in other domains, e.g. Degrave et al. (2022) who leveraged simulation and experimental data to learn a closed-loop controller a tokamak reactor. Recent research explores similar hybrid approaches to control plant and crop systems (Kang and Wang, 2017; Ifrim et al., 2021; Mahmood et al., 2023). Simulation and scientific computing is centered around creating mechanistic computational models to simulate real-world phenomena, while machine learning focuses on leveraging learning algorithms to extract knowledge and insights from scientific data. Both approaches have strengths and can be combined to enhance scientific understanding and decision-making.

Throughout our literature survey, we identified the SI motifs embedded in numerous plant-related projects. This finding aligns with the core objectives of SI, which are geared towards addressing the issues inherent in modeling complex systems:

- Solving inverse problems;
- Integrating mechanistic knowledge with data;
- Navigating uncertainty, and;
- Fostering effective communication between the model and the user.

In applied domains, such as plant and crop modeling, advancements often trail the cutting edge of computational methodologies. Consequently, it is unsurprising that relatively mature SI motifs, such as surrogate models, agent-based modeling, and differentiable programming, showcase the highest prevalence in plant-related examples. In contrast, motifs in their infancy, such as probabilistic programming, open-ended optimization, and program synthesis, exhibit fewer concrete applications in plant and crop science. These emerging SI topics can advance plant and agricultural sciences toward a more sustainable future.

## Data availability statement

The original contributions presented in the study are included in the article/supplementary material. Further inquiries can be directed to the corresponding author.

## Author contributions

MS: Writing – original draft, Writing – review & editing. OP: Writing – original draft, Writing – review & editing. TD: Writing – original draft, Writing – review & editing. FW: Writing – original draft, Writing – review & editing.

## Footnotes

1
https://quantitative-plant.org/(accessed on March 2023).
